# Editorial: Social commerce in the new era

**DOI:** 10.3389/fpsyg.2022.1010357

**Published:** 2022-09-13

**Authors:** Shuiqing Yang, Yuan Sun, Atika Qazi, Jiabao Lin, Khalid Haruna

**Affiliations:** ^1^School of Information Management and Artificial Intelligence, Zhejiang University of Finance and Economics, Hangzhou, China; ^2^College of Business Administration, Zhejiang Gongshang University, Hangzhou, China; ^3^Centre for Lifelong Learning, Universiti Brunei Darussalam, Bandar Seri Begawan, Brunei; ^4^School of Economics and Management, South China Agricultural University, Guangzhou, China; ^5^Department of Computer Science, Kaduna State University, Kaduna, Nigeria

**Keywords:** social commerce, social media, livestream shopping, consumers' online review, metaverse

Social commerce in the new era, a time encompassing the COVID-19 pandemic and the widespread application of artificial intelligence, presents new phenomena, raising new Research Topics and questions for researchers. This Research Topic is aimed at enhancing and widening knowledge on social commerce in the new era. The Research Topic includes seven papers from several fields across social commerce purchases and repurchases intentions, including online crowdsourcing vendors' market participation rate and market revenue share, and users' behaviors toward bike-sharing and intelligent vehicles.

Three papers examine Research Topics related to social commerce purchases and repurchase intentions. Based on para-social interaction theory, Zhu et al. explore how the characteristics of anchors may influence consumer behavioral intentions in livestream shopping. They found that the anchors' physical attractiveness, social attractiveness, and professional ability affect whether consumers follow the anchors' suggestions and recommend the use of anchors during livestreams *via* para-social interaction and affective trust in anchors. This study applied para-social interaction theory in the context of new media and validated it as a suitable theoretical foundation to explain the livestream shopping behaviors of consumers. Similarly, considering how the COVID-19 pandemic has had a significant impact on the retail industry, Alam et al. examined the involvement of social media influencers in social commerce and the mediating effect of community trust on social commerce intentions and online purchasing intention in India. They found that the features of social media influencers enhance users' trust in the online community and social commerce intention, which in turn affects their online purchasing intentions. Based on the stimulus-organism-response framework, Guo and Li examine the impact of social commerce features on consumers' value perceptions and repurchase intentions. They found that social commerce features including interactivity, recommendations, and feedback positively affect perceived utilitarian value and hedonic value, which in turn positively affect repurchase intentions. The COVID-19 pandemic has greatly impacted the retail industry.

One paper studied the sustainable performance of online crowdsourcing vendors. Drawing on transaction cost theory, Han et al. explore the influences of reputation growth on online crowdsourcing vendors' market participation rate and market revenue share. Based on fixed-effect panel data regression analyses, this study found that vendors' reputation exerts a negative impact on participation rate in both task-based markets and employment markets. Reputation also has a consistent influence on the vendors' revenue share of each market.

Two papers studied Research Topics related to user behaviors toward bike-sharing and intelligent vehicles. Based on the theory of planned behavior, Chen examined the impact of perceived benefits and government policy on college students' bike-sharing intentions. This study found that government policy and perceived benefits combined with subjective norms, perceived behavioral control, and attitude were the important factors driving college students' intention and behavior for bike-sharing usage. Chen's work enhances current understanding of the usage behavior of college students when bike-sharing. Through the lens of psychological empowerment theory, Li et al. identify three dimensions of users' perceived empowerment (perceived cognitive empowerment, perceived emotional empowerment, and perceived behavioral empowerment). They found that the perceived empowerment of drivers is vital for their willingness to use automatic features of intelligent vehicles.

One paper studied Research Topics related to online review and consumption behaviors. Based on cue utilization theory, Xiao et al. examined how the review party's cues may affect consumers' adoption willingness and consumption behavior. The results of an event-related potential experiment showed that the review party's cues, including professionalism and popularity influence consumers' evaluation and classification process.

The papers published in this Research Topic provide a good reference for scholars interested in studying social commerce. Considering the rapid evolution of social media (e.g, the metaverse), we hope that more scholars will be attracted to explore these emerging research issues of social commerce in the new era.

## Author contributions

SY and AQ: writing—original draft. YS, JL, and KH: writing—review and editing. All authors contributed to the article and approved the submitted version.

## Conflict of interest

The authors declare that the research was conducted in the absence of any commercial or financial relationships that could be construed as a potential conflict of interest.

## Publisher's note

All claims expressed in this article are solely those of the authors and do not necessarily represent those of their affiliated organizations, or those of the publisher, the editors and the reviewers. Any product that may be evaluated in this article, or claim that may be made by its manufacturer, is not guaranteed or endorsed by the publisher.

